# Suicide attempts in women with severe mental illness in the perinatal period

**DOI:** 10.1192/j.eurpsy.2021.143

**Published:** 2021-08-13

**Authors:** F. Gressier, A.-L. Sutter-Dallay

**Affiliations:** 1 Department Of Psychiatry, Bicetre Hospital, CESP, University Paris-Saclay, University Paris-Sud, Le Kremlin Bicetre, France; 2 Univ. Bordeaux, Inserm, Bph, U1219, F-33000 Bordeaux, France And Perinatal Psychiatry Network-university Department Of Child And Adolescent Psychiatry, Bordeaux University and Charles Perrens Hospital, Bordeaux Cedex, France

**Keywords:** Mental Illness, Suicide Attempt, pregnancy, post-partum

## Abstract

Suicide is one of the leading causes of perinatal maternal mortality (1). Maternal suicidality has a negative impact on the mother-baby relationship and child development. However, little is known about specific risk factors for perinatal suicide attempts in women with severe mental illness. In a sample of 1439 women with severe mental illness in the perinatal period and jointly admitted with their baby in a mother and baby unit, 154 (11.7%) attempted suicide, 49 in pregnancy (3.7%) and 105 (8.0%) in the post-partum period (2). Suicide attempt in pregnancy was related to alcohol use, smoking during pregnancy and a history of miscarriage, and in the post-partum period to major depressive episode or recurrent depression and younger age. Women who attempt suicide either in pregnancy or in the postnatal period could have different psychopathological and environmental profiles. Past obstetric history and addictive behaviours during pregnancy are essential elements to explore. In addition, depressive symptoms should be assessed in all women to treat major depression, as a means of preventing suicide attempt. Special attention to risk of suicide is needed during the perinatal period for women with severe mental illness. For women suffering from an acute psychiatric disorder, or a history of mental illness, multi-disciplinary management should be implemented. 1. Oates M. Suicide: the leading cause of maternal death. Br J Psychiatry. 2003;183:279-81. 2. Gressier F et al. Risk factors for suicide attempt in pregnancy and the post-partum period in women with serious mental illnesses. J Psychiatr Res. 2017;84:284-291.

**Disclosure:**

No significant relationships.

